# Sex, body mass index, and blood pressure are related to aortic characteristics in healthy, young adults using magnetic resonance vessel wall imaging: the AMBITYON study

**DOI:** 10.1007/s10334-017-0626-z

**Published:** 2017-05-31

**Authors:** Anouk L. M. Eikendal, Hester M. den Ruijter, Cees Haaring, Tobias Saam, Rob J. van der Geest, Jos J. M. Westenberg, Michiel L. Bots, Imo E. Hoefer, Tim Leiner

**Affiliations:** 1 Department of Radiology (E01.132), University Medical Center Utrecht, Heidelberglaan 100, 3584 CX Utrecht, The Netherlands; 2 Laboratory of Experimental Cardiology, University Medical Center Utrecht, Heidelberglaan 100, 3584 CX Utrecht, The Netherlands; 3 Institute of Clinical Radiology, Ludwig-Maximilians-University Hospital, Marchioninistrasse 15, 81377 Munich, Germany; 4 Division of Image Processing, Department of Radiology, 1-C2S Leiden University Medical Center, PO Box 9600, 2300 RC Leiden, The Netherlands; 5 Julius Center for Health Sciences and Primary Care, University Medical Center Utrecht, Heidelberglaan 100, 3584 CX Utrecht, The Netherlands; 6 Laboratory of Clinical Chemistry and Hematology, University Medical Center Utrecht, Heidelberglaan 100, 3584 CX Utrecht, The Netherlands

**Keywords:** MRI, Risk factors, Health, Young adult

## Abstract

**Objectives:**

More detailed evaluation of atherosclerosis and its key determinants in young individuals is warranted to improve knowledge on the pathophysiology of its development and progression. This study evaluated associations of magnetic resonance imaging (MRI)-derived aortic wall area, wall thickness, and pulse wave velocity (PWV) with cardiovascular risk factors in asymptomatic, young adults.

**Materials and methods:**

In 124 adults (age: 25–35 years) from the general population-based Atherosclerosis Monitoring and Biomarker Measurements in the Young study, demography, anthropometry, and blood samples were collected. The studied MRI-parameters were measured using a 3.0T MRI system. Relations between cardiovascular risk factors and aortic characteristics were assessed using multivariable linear regression analyses.

**Results:**

Mean age was 31.8 years, 47.6% was male. Aortic wall area was positively associated with age [*β* = 0.01, (95% confidence interval (CI) 2.00 × 10^−3^, 0.02), *p* = 0.01] and BMI [*β* = 0.01, (0.01, 0.02), *p* = 0.003] and negatively associated with sex (reference: men) [*β* = −0.06, (−0.11, −0.01), *p* = 0.02]. Natural logarithm transformed (ln) aortic wall thickness was positively associated with BMI [*β* = 0.01, (1.00 × 10^−3^, 0.02), *p* = 0.02]. Ln aortic PWV was positively associated with 10 mmHg increment of SBP [*β* = 0.06, (0.03, 0.09), *p* < 0.001] and DBP [*β* = 0.06, (0.02, 0.09), *p* = 0.006]. No relations were observed for smoking and lipids.

**Conclusions:**

Already in early adulthood, aortic wall geometry and stiffness vary by age, sex, BMI, and blood pressure.

## Introduction

The incapacitating sequelae of atherosclerosis remain a major contributor to the global burden of disease [1]. Hence, prevention of symptomatic atherosclerosis remains imperative. Atherosclerosis is a generalized inflammatory disease that prompts unfavorable arterial wall remodeling from childhood onwards, yet remains clinically dormant for years before developing into clinically overt cardiovascular disease (CVD) [2].

Its pattern of disease enables preclinical detection of atherosclerosis. Given that CVD risk rises due to a lengthy and cumulative exposure to adverse levels of amenable CV risk factors that are already highly prevalent from a young age onwards, early detection of atherosclerosis is warranted. This may allow for early identification of high-risk individuals and thus may enable effective treatment of factors related to atherosclerosis extent and progression in these individuals. In order to achieve this, more detailed understanding of the development and progression of atherosclerosis in young individuals is warranted.

In this view, assessment of the presence and extent of aortic atherosclerosis using magnetic resonance imaging (MRI) may be promising. The aorta is an attractive target for the assessment of atherosclerosis in early life since it is one of the first arteries to be affected by atherosclerosis and the artery where atherosclerosis is most manifest [2, 3]. MRI has evolved as an attractive modality for in vivo evaluation of aortic atherosclerosis in various populations since it allows for direct evaluation of the target organ deep within the body, is non-invasive and non-ionizing. Furthermore, MRI is able to cover a large anatomical territory, has superior soft tissue contrast and can easily be repeated sequentially over time [4]. Changes in MRI-derived aortic characteristics such as wall thickness, plaque burden, and pulse wave velocity (PWV) have consistently been related to known CV risk factors and CV events [4–10].

Although MRI is a particularly attractive tool for evaluation of the subtle aortic wall alterations related to atherosclerosis, data on this matter in young adults are very limited [11]. Therefore, the objective of the present study was to explore the relation of known CV risk factors with MRI-derived early signs of atherosclerosis such as aortic wall area, wall thickness, and PWV in a population of healthy, young adults.

## Materials and methods

### Study design and study population

The general population-based Atherosclerosis Monitoring and Biomarker Measurements in the Young (AMBITYON) study (Netherlands National Trial Register number: 4742) is a prospective, mono-center cohort study that aimed to evaluate the interrelation between classical CV risk factors, blood biomarkers of arterial inflammation and MRI-derived atherosclerosis burden in young adulthood. Study rationale and detailed description were published recently [12]. In short, the AMBITYON study aspires to expand current knowledge of the pathophysiology of development and progression of atherosclerosis by further clarifying key determinants of symptomatic CVD later in life. To date, 131 participants have been included in the AMBITYON study. Individuals were recruited from Leidsche Rijn, a district in Utrecht city, the Netherlands. To be suitable for enrolment in the AMBITYON study, individuals had to be aged between 25 and 35 years and free from (a history of) symptomatic CVD, use of CV protective medication, cardiac arrhythmias, and contra-indications to MRI (i.e. pregnancy). The AMBITYON study was granted approval from the institutional review board (IRB) of the University Medical Center (UMC) Utrecht (IRB: 13/397). From each participant, written informed consent was obtained before enrolment.

### Demographic information

All AMBITYON study participants filled out a detailed and standardized electronic questionnaire that included inquiries on the participant’s demographic profile and health status. With this questionnaire, information on CV lifestyle risk factors was acquired [13–15].

### Anthropometric measurements

Before the MRI examination, body height and weight were measured in all participants using a stadiometer and weighing scale, respectively. The participants were clothed in an indoor outfit without shoes and placed in a standing position with the feet somewhat apart. Waist and hip circumference were measured halfway between the iliac crest and the most distal rib border and at the widest position over the major trochanter, respectively. Body mass index (BMI; kg/m^2^) was calculated using height and weight data. Blood pressure measurements were obtained using an automated oscillometric device with adult cuffs (Welch Allyn, NY, USA). Blood pressure was measured twice at the right brachial artery after 5 min of rest with the participant in a sitting position and a time interval of 5 min between each measurement. Mean systolic (SBP) and diastolic (DBP) blood pressure were calculated as the average of both measurements, respectively.

### Aortic characteristics

#### MRI protocol

All participants underwent an MRI examination of the aorta in supine posture on a 3.0T multi-transmit clinical MRI system (Achieva, Software Release 5.1.7.2, Philips Healthcare, Best, the Netherlands). Imaging was performed using a 32-channel phased-array cardiac receiver coil that combines two flexible 16-element coils positioned anterior and posterior to the participant. The posterior part of the coil is integrated in a housing mattress embedded on the MRI table top. The MRI examination was executed within standard limits of the specific absorption rate (SAR). Before the MRI examination, each participant was coached to perform end-expiratory breath holding. Total MRI examination time was circa 60 min per participant.

#### Assessment of aortic wall geometry and pulse wave velocity

A detailed description of the measurements as mentioned below, including the MRI assessment and image analysis of aortic wall geometry, thickness, and PWV can be found elsewhere [12]. In short, for measurement of aortic wall area and thickness, images of the descending thoracic aorta were acquired in the sagittal orientation using a 3-dimensional (3D) black-blood (BB), T1-weighted, turbo spin echo (TSE) sequence with variable flip angles (3D-T1-BB-VISTA), a sensitivity encoding (SENSE) parallel imaging algorithm and spectral attenuated inversion recovery (SPAIR) fat suppression. Images were acquired during free breathing without ECG gating. The black-blood effect was realized by intrasequence flow related dephasing. The field of view (FOV; 350 × 302 × 45 mm) was placed between the top of the aortic arch and the most distal boundary of the cardiac coil, spanning approximately 35 cm of descending thoracic aorta. This FOV enabled full coverage of the thoracic descending aorta and a small segment of the abdominal aorta.

To assess stiffness of the thoracic aorta, global PWV was measured over the whole thoracic aorta as described previously [12, 16]. In short, a single-slice, double oblique, turbo field gradient-echo survey image was acquired in the sagittal orientation with retrospective ECG gating and a single end-expiratory breath hold to visualize the full course of the thoracic aorta. Based on this image, two phase contrast acquisitions with velocity-encoding (VE) were planned at right angles to the aortic center lumen line to measure the through-plane flow velocity in the ascending and proximal descending thoracic aorta (acquisition 1) and in the distal descending thoracic aorta (acquisition 2). Flow measurements were acquired in the transversal orientation using a one-directional, through-plane, non-segmented, VE (1.50 m/s), gradient turbo field echo pulse sequence with retrospective ECG gating during free breathing. Over one cardiac cycle, 50 heart phases were reconstructed (temporal resolution of 10–20 ms depending on heart rate, TFE shot duration: 38.6 ms; actual temporal resolution: 77.2 ms), interpolation: 50%).

#### MR image analysis

**Fig. 1:**
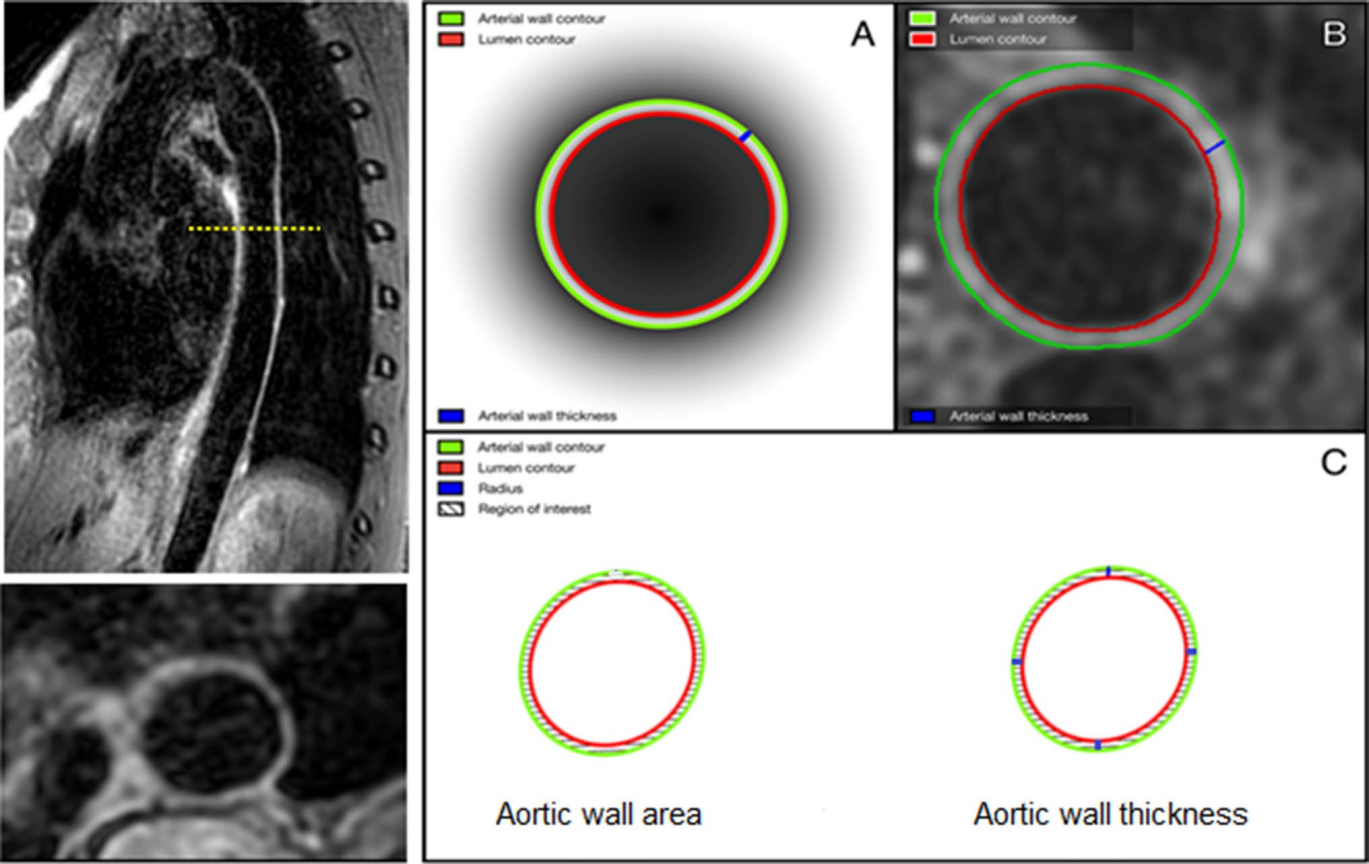
Illustration of 3D-T1-BB-VISTA sequence and measurement of aortic wall geometry (32-year-old female participant). *Left* illustration of sagittal and transversal (reconstructed) images of the descending thoracic aorta acquired using the 3D-T1-BB-VISTA sequence. *Right* illustrations of quantification methods of aortic wall area and wall thickness. **a**, **b** A schematic and in vivo example of tracing of the luminal and outer contours, as well as an aortic wall thickness measurement. **c** A graphic illustration of the method of quantification of aortic wall area and wall thickness. Each zone between two *blue lines* embodies an aortic wall section. In each section, 25 thickness measurements are performed, generating in total 100 thickness measurements per image

Aortic wall area and thickness were quantified with a validated software program exclusively equipped for measuring MRI-derived arterial characteristics [Vessel Mass, release 5.1, Laboratory for Clinical and Experimental Image processing (LKEB), the Netherlands] [11]. Image analysis was carried out according to a standardized protocol [12]. Because of the restricted craniocaudal coverage of the reception coil, the aorta was analyzed between the beginning of the descending thoracic aorta and the beginning of the celiac trunk. Aortic wall characteristics were measured in one image per centimeter of craniocaudal coverage. On average 22 cm of descending thoracic aorta was analyzed per participant. In the present study, 52 out of 2928 images (1.8%, conforming to one image in 52 participants) that were used to quantify aortic wall thickness and wall area had an insufficient image quality; therefore, these images were omitted from analysis. Vessel mass computed the mean aortic wall area and thickness in each participant by summing the mean aortic wall area (cm^2^) and thickness (mm) for all analyzed images in that participant and dividing it by the number of analyzed images in that participant. An example of the 3D-T1-BB-VISTA acquisition and a graphic illustration of aortic wall area and thickness quantification is displayed in Fig. 1. In a prior study, we demonstrated that inter-scan, as well as inter- and intra-rater reproducibility of aortic wall measurements using the 3D-T1-BB-VISTA sequence was excellent [intra-class correlation coefficient (ICC) 0.76-0.99] [12].

**Fig. 2:**
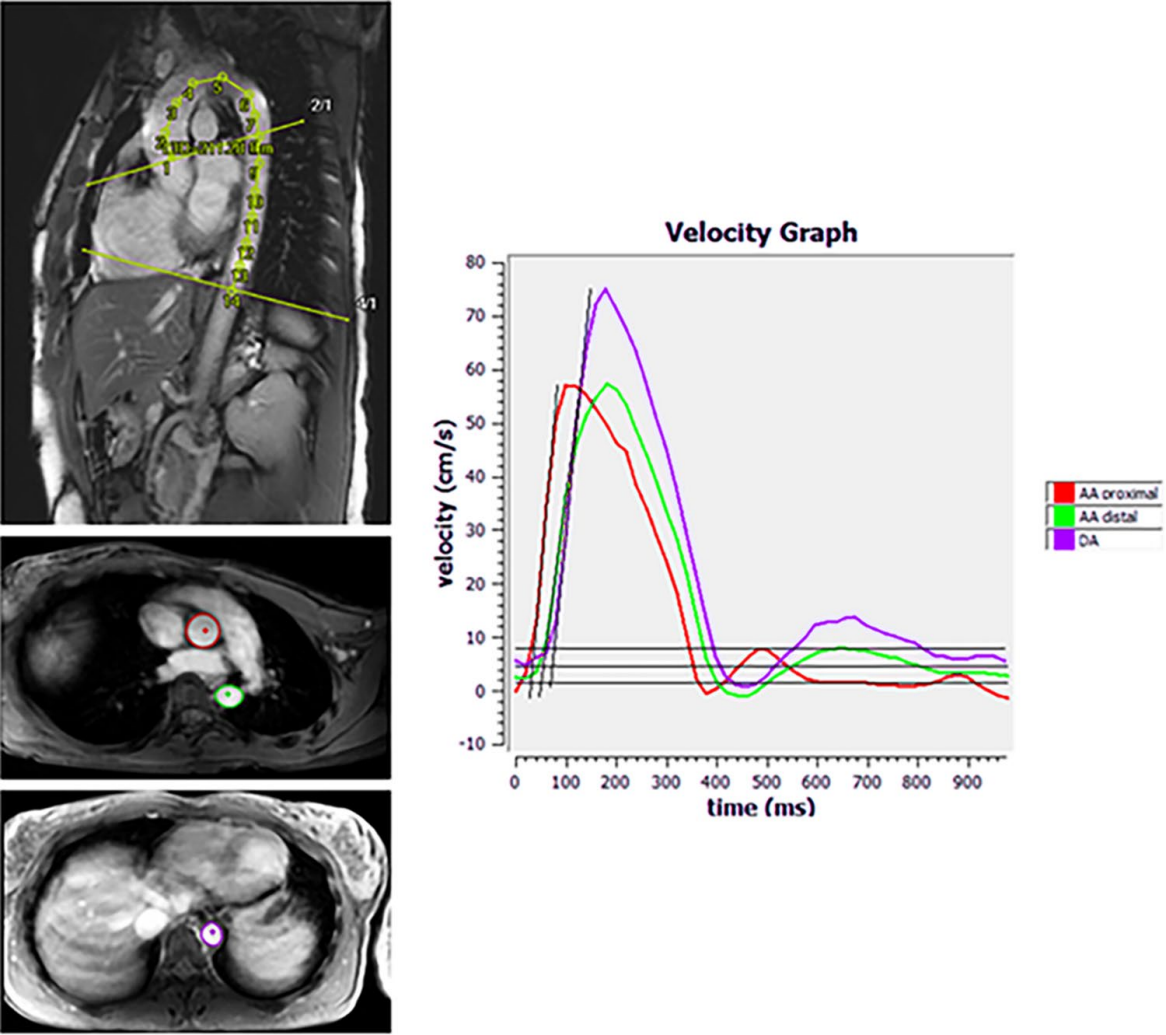
Example of double oblique, through-plane velocity-encoded images and of PWV measurement (32-year-old female participant). *Upper left* illustration of traced double oblique image. Tracing was performed along the centerline of the aorta to measure aortic lengths. *Middle/lower left* illustration of traced through-plane images. Contours were traced in the ascending and proximal descending aorta (acquisition 1 *middle left*) and near the dome of the liver (acquisition 2 *lower left*) for aortic velocity mapping. *Right* example of velocity graph, generated using PWVAppStatic. PWV of the total thoracic aorta was measured using linear modelling

Aortic PWV was computed as *X*/*t* (m/s). *X* is the aortic length between the three measurement locations as described above; *t* is the transit time between the arrival of the systolic pulse wave front at each of these locations. Image analysis was performed conforming to a standardized procedure [17]. In short, to assess PWV, aortic length between each measurement location was obtained by manually tracing the aorta along its centerline in the double oblique image using MASS software [12, 16]. Next, with a semi-automatic flow analysis tool in MASS, the outer contours of the ascending and proximal descending thoracic aorta (acquisition 1), as well as the distal descending thoracic aorta (acquisition 2) in all 50 heart phases were traced. As such, aortic velocity maps were generated. These were further analyzed using a validated PWV measurement software program (PwvAppStatic, LKEB, Leiden, the Netherlands). The PwvAppStatic uses the aortic velocity maps to create velocity–time curves and calculates the transit time between the measurement locations from these curves. By combining the results of the aortic length measurements and transit time absolute PWV values for the total thoracic aorta were generated using linear regression modelling. An example of the double oblique and VE images, as well as an example of the PWV measurement method is displayed in Fig. 2. PWV was quantified according to a widely used, highly reproducible and validated method with excellent inter-scan, inter-rater, and intra-rater reproducibility (ICC: 0.87–0.92) [16, 18].

In 7/131 (5.3%) participants, aortic imaging failed due to technical malfunction of the cardiac coil. Also, in 6/124 (4.8%) participants in whom aortic imaging succeeded, image quality was considered insufficient for aortic PWV quantification. Moreover, in two of 124 (1.6%) remaining participants, venous puncture was unsuccessful. Hence, for laboratory demographics, aortic wall characteristics and aortic PWV, complete case multivariable analysis was performed in 122/131 (93.1%), 124/131 (94.7%), and 118/131 (90.1%) participants, respectively.

### Laboratory assessments

In each participant, a venous blood sample was obtained in two tubes: a sodium-heparin tube (3 mL) and EDTA tube (2 mL). Immediately after collection, the Laboratory of Clinical Chemistry of the UMC Utrecht processed the sample and measured total cholesterol, HDL-cholesterol, glucose, triglyceride, and C-reactive protein (CRP) levels using a routine clinical chemistry analyzer (AU5811, Beckman Coulter, Brea, CA, USA). The LDL-cholesterol level was computed using the Friedewald formula [19]. Moreover, white blood cell count (WBC) was measured with a hematology analyzer (CellDyn Sapphire, Abbott, Chicago, IL, USA). Of note, for CRP levels below detection limit (0.5 mg/L) the CRP was estimated by using the mean level between zero and the lower detection limit.

### Data analysis

Demographic characteristics of the participants were presented at the participant level as means with standard deviations (SD) or medians with 25th and 75th percentiles (Q1 and Q3) if they were continuous and had a normal or non-normal distribution, respectively. Normality of distribution of each variable was tested using the Shapiro–Wilk test of normality for relatively small sample sizes, histograms, and QQ-plots. Categorical characteristics were presented as numbers and percentages.

The relations of CV risk factors (independent variables) with mean aortic wall area (cm^2^), wall thickness (mm), and mean aortic PWV (m/s) (dependent variables) were assessed using linear regression analysis. The studied CV risk factors were age, sex (reference category: men), smoking (current versus never and former versus never, reference category: never), BMI (kg/m^2^), SBP (mmHg), DBP (mmHg), total, HDL and LDL cholesterol level (all in mmol/L), and the Framingham Risk Score (FRS) [20]. The FRS, a composite score, was calculated for each participant by creating risk categories for each FRS risk factor according to the FRS cut-off points and consecutively summing the points acquired for each risk factor [20]. Two linear regression models were constructed. First, a crude model (Model 1) was created in which the unadjusted associations of the studied CV risk factors with the studied aortic characteristics were evaluated. Subsequently, a multivariable model (Model 2) was created, in which we adjusted Model 1 for the following a priori selected confounders: smoking, BMI, DBP, total, and HDL cholesterol level. Because of a high correlation between SBP and DBP (Spearman’s *r* = 0.75) and LDL and total cholesterol (Spearman’s *r* = 0.89) we did not adjust SBP for DBP and LDL for total cholesterol. Since the FRS is an accumulated measure of CV risk, we did not adjust the FRS for any of the included variables.

To meet the mandatory criteria for linear regression analyses, mean aortic wall thickness, and PWV were transformed using natural logarithm transformation (ln) to normalize skewed distributions. Conclusions were based on linear regression coefficients (*β*) with 95% confidence intervals (CI) and *p* values. A two-sided *p* value of <0.05 was considered statistically significant. Data analysis was performed using SPSS version 21.0 (IBM, Armonk, NY, USA).

## Results

### Demographic characteristics

Demographic characteristics of the AMBITYON study population are summarized in Table 1. Median age of the 124 participants under study was 31.8 years (29.1, 34.1); 59 participants (47.6%) were male. Mean aortic wall area was 1.0 cm^2^ (±0.1) whereas median aortic wall thickness and PWV were 1.5 mm (1.4, 1.7) and 4.4 m/s (4.1, 4.8), respectively.

**Table 1:** Characteristics of study population

	*N*	Total population
**Demographic characteristics**		
Age (years), median (Q1, Q3)^a^	124	31.8 (29.1, 34.1)
Sex (men), *n* (%)	124	59 (47.6)
Current cigarette smoking (yes), *n* (%)	124	27 (21.8)
Former cigarette smoking (yes), *n* (%)	124	23 (18.5)
Diabetes mellitus (yes), *n* (%)	124	1 (0.8%)
**Anthropometric characteristics**		
Height (cm), mean (SD)^a^	124	176.6 (8.8)
Weight (kg), mean (SD)^a^	124	73.5 (11.6)
Waist circumference (cm), mean (SD)^a^	124	79.6 (8.8)
Hip circumference (cm), median (Q1, Q3)^a^	124	87.0 (82.0, 92.0)
BMI (kg/m^2^), median (Q1, Q3)^a^	124	23.2 (21.6, 25.0)
SBP (mm Hg), mean (SD)^a^	124	128.0 (12.0)
DBP (mm Hg), mean (SD)^a^	124	79.0 (8.0)
**Laboratory assessments**		
Total cholesterol level (mmol/L), mean (SD)^a^	122	4.6 (0.8)
HDL-cholesterol level (mmol/L), median (Q1, Q3)^a^	122	1.4 (1.2, 1.7)
LDL-cholesterol level (mmol/L), mean (SD)^a^	122	2.6 (0.7)
Triglyceride level (mmol/L), median (Q1, Q3)^a^	122	1.2 (0.9, 1.8)
Glucose level (mmol/L), median (Q1, Q3)^a^	122	5.1 (4.7, 5.5)
**MRI derived aortic characteristics**		
Mean aortic wall area (cm^2^), mean (SD)^a^	124	1.0 (0.1)
Mean aortic wall thickness (mm), median (Q1, Q3)^a^	124	1.5 (1.4, 1.7)
Pulse wave velocity (m/s), median (Q1, Q3)^a^	118	4.4 (4.1, 4.8)

^a^ *Q1* 25th percentile, *Q3* 75th percentile, *SD* standard deviation, *BMI* body mass index, *SBP* systolic blood pressure, *DBP* diastolic blood pressure, *HDL* high-density lipoprotein, *LDL* low-density lipoprotein

### Aortic wall geometry

The results of the relation of CV risk factors with aortic wall characteristics are listed in Table 2. The multivariable linear regression model (Model 2) showed a positive relation between mean aortic wall area and age [*β* = 0.01, (95% CI: 2.00 × 10^−3^, 0.02), *p* = 0.01] and BMI [*β* = 0.01, (95% CI: 0.01, 0.02), *p* = 0.003] and a negative relation between mean aortic wall area and sex [*β* = −0.06 (reference category: men), (95% CI: −0.11, −0.01), *p* = 0.02]. Ln-mean aortic wall thickness was positively related to BMI [*β* = 0.01, (95% CI: 1.00 × 10^−3^, 0.02), *p* = 0.02]. We did not observe a relation between age and sex and aortic wall thickness, nor did we observe a relation of any of the other CV risk factors under study or FRS with aortic wall geometry.

**Table 2:** Relation between classical cardiovascular risk factors and aortic characteristics

	Aortic wall area (cm^2^)^a^ (*n* = 124)	*p* value	Aortic wall thickness (mm)^ab^ (*n* = 124)	*p* value	Aortic PWV (m/s)^ab^ (*n* = 118)	*p* value
**Age, per year increase** ^c^						
Model 1	0.01 (5.00 × 10^−3^, 2.00)	0.001^§^	3.00 × 10^−3^ (−3.00 × 10^−3^, 0.01)	0.34	−3.00 × 10^−4^ (−0.01, 9.00 × 10^−3^)	0.95
Model 2	0.01 (2.00 × 10^−3^, 0.02)	0.01^§^	2.00 × 10^−3^ (−4.00 × 10^−3^, 9.00 × 10^−3^)	0.48	−1.00 × 10^−4^ (−0.01, 9.00 × 10^−3^)	0.99
**Sex (reference: men)** ^c^						
Model 1	−0.07 (−0.11, −0.03)	0.002^§^	0.02 (−0.02, 0.06)	0.44	−0.01 (−0.07, 0.05)	0.70
Model 2	−0.06 (−0.11, −0.01)	0.02^§^	0.02 (−0.03, 0.07)	0.39	−0.01 (−0.08, 0.05)	0.72
**Current smoking (reference: never)** ^c^						
Model 1	−0.01 (−0.07, 0.04)	0.65	0.01 (−0.04, 0.06)	0.67	0.02 (−0.05, 0.09)	0.60
Model 2	−0.03 (−0.08, 0.03)	0.37	−1.00 × 10^−3^ (−0.05, 0.05)	0.97	0.01 (−0.06, 0.09)	0.70
**Former smoking (reference: never)** ^c^						
Model 1	0.07 (0.02, 0.13)	0.01	0.01 (−0.04, 0.06)	0.74	0.02 (−0.04, 0.09)	0.48
Model 2	0.04 (−0.02, 0.10)	0.18	0.02 (−0.05, 0.06)	0.93	0.03 (−0.05, 0.09)	0.57
**BMI, per kg/m** ^**2**^ ** increase** ^cd^						
Model 1	0.01 (0.01, 0.02)	0.001^§^	0.01 (1.00 × 10^−3^, 0.02)	0.03^§^	0.01 (−1.00 × 10^−3^, 0.02)	0.09
Model 2	0.01 (0.01, 0.02)	0.003^§^	0.01 (1.00 × 10^−3^, 0.02)	0.02^§^	2.00 × 10^−4^ (−0.01, 0.01)	0.72
**SBP, per 10 mm Hg increase** ^cde^						
Model 1	0.02 (3.00 × 10^−3^, 0.04)	0.02^§^	0.01 (−0.01, 0.03)	0.19	0.05 (0.03, 0.07)	<0.001^§^
Model 2	3.00 × 10^−3^ (−0.02, 0.02)	0.74	0.01 (−0.01, 0.03)	0.23	0.06 (0.03, 0.09)	<0.001^§^
**DBP, per 10 mm Hg increase** ^cd^						
Model 1	0.01 (−0.02, 0.04)	0.42	0.01 (−0.02, 0.04)	0.45	0.06 (0.02, 0.09)	0.001^§^
Model 2	−0.01 (−0.04, 0.02)	0.57	2.00 × 10^−3^ (−0.03, 0.03)	0.88	0.06 (0.02, 0.09)	0.006^§^
**Total cholesterol, per mmol/L increase** ^c^						
Model 1	0.01 (−0.02, 0.04)	0.35	4.00 × 10^−3^ (−0.02, 0.03)	0.74	0.02 (−0.02, 0.05)	0.31
Model 2	−0.01 (−0.04, 0.02)	0.60	−0.01 (−0.04, 0.02)	0.42	0.01 (−0.03, 0.05)	0.77
**HDL cholesterol, per mmol/L increase** ^cd^						
Model 1	−0.04 (−0.10, 0.03)	0.30	0.02 (−0.04, 0.08)	0.59	0.02 (−0.07, 0.11)	0.66
Model 2	0.03 (−0.42, 0.11)	0.39	0.03 (−0.05, 0.10)	0.50	0.04 (−0.06, 0.14)	0.39
**LDL cholesterol, per mmol/L increase** ^cde^						
Model 1	3.00 × 10^−3^ (−0.03, 0.04)	0.87	1.00 × 10^−3^ (−0.03, 0.03)	0.93	2.00 × 10^−3^ (−0.04, 0.04)	0.92
Model 2	−0.02 (−0.05, 0.01)	0.25	−0.01 (−0.04, 0.02)	0.48	−2.00 × 10^−3^ (−0.05, 0.04)	0.94
**Framingham Risk Score, per point increase** ^c^						
Model 1	3.00 × 10^−4^ (−4.00 × 10^−3^, 0.01)	0.89	2.00 × 10^−3^ (−2.00 × 10^−3^, 0.01)	0.24	2.00 × 10^−3^ (−4.00 × 10^−3^, 0.01)	0.50

^§^ *p* < 0.05

^a^Values are linear regression coefficients (β) with 95% confidence intervals

^b^Natural logarithmic transformation was performed

^c^Model 1: crude model, Model 2: adjusted for age, sex, smoking, BMI, DBP, total cholesterol, and HDL cholesterol

^d^ *BMI* body mass index, *SBP* systolic blood pressure, *DBP* diastolic blood pressure, *HDL* high-density lipoprotein, *LDL* low-density lipoprotein

^e^SBP was not adjusted for DBP and LDL cholesterol was not adjusted for total cholesterol

### Aortic pulse wave velocity

The results of the relation of CV risk factors with ln-mean aortic PWV are listed in Table 2. The multivariable linear regression model (Model 2) showed a positive relation between ln-mean aortic PWV and 10 mmHg increment of SBP [*β* = 0.06, (95% CI: 0.03, 0.09), *p* < 0.001] and DBP [*β* = 0.06, (95% CI: 0.02, 0.09), *p* = 0.006]. We did not observe a relation of the other CV risk factors under study and FRS with aortic PWV.

Of note, additional analyses with aortic characteristics dichotomized into ≥75th percentile and <75th percentile and additional analyses with CV risk factor levels dichotomized into 75th percentile and <75th percentile did not change our results.

## Discussion

This study investigated the relation between CV risk factors and evidence of early atherosclerosis and aortic wall stiffness using aortic MRI in young adults without known cardiovascular disease. We observed associations of CV risk factors with alterations in aortic wall geometry (age, sex, and BMI) and aortic stiffness (SBP and DBP) already in young adulthood. To the best of our knowledge, this is the first study to report these associations in an asymptomatic young population using MRI-derived measures for early subclinical atherosclerosis. Using a highly innovative imaging technique, this study confirms current knowledge on the relation of CV risk factors with subclinical atherosclerosis to young adults in whom atherosclerosis related arterial alterations are still subtle, and the opportunity for primary prevention is still present.

The aortic wall is not a rigid conduit, but a biologically dynamic integrated organ that harbors smooth muscle cells, endothelial cells, elastin, collagen, and fibroblasts, is able to notice hemodynamic stimuli and releases vasoactive elements [21]. Its structure and function are affected by various (un)known pathophysiological factors [3, 21]. Accurate evaluation of the arterial wall in young individuals permits mapping of its morphology and function and may uncover the effects of these factors on its bed. This may further unravel the pathophysiology of atherosclerosis and reveal factors that influence its development and progression. Ultimately, this may provide information to improve pre-clinical identification of asymptomatic, young individuals who are at high-risk for developing clinically overt CVD later in life and may enable early initiation of targeted therapy in these individuals.

As an entirely non-invasive imaging modality, MRI-derived imaging markers for aortic atherosclerosis may suit this purpose. Prior studies have assessed the relation of CV risk factors with structural and functional MRI-derived aortic characteristics in asymptomatic and CV diseased populations [5–7, 9]. Most of these studies were less comprehensive and mostly incorporated middle to older aged individuals. These studies showed that structural and functional aortic characteristics relate to CV risk factors and to CV events. Mean thoracic aortic wall area has been related to coronary heart disease (CHD) and to CV events [21–23]. In addition, descending thoracic aortic mean wall thickness increases with age, male sex, current smoking, higher BMI, higher SBP, and an unfavorable lipid profile [5–7, 9]. From a functional perspective, aortic PWV has shown to be an important predictor of CV events and CHD [8, 10, 16, 24, 25]. PWV predominantly increases with an older age and to a lesser extent with a higher SBP, higher DBP, higher BMI, abnormal lipid profile, and impaired glucose metabolism [8].

The present results are partially in agreement with prior studies. In our study, mean aortic wall area was related to age, sex, and BMI. Aortic wall area offers information on pathophysiological processes that regulate growth and regression of the arterial structure. Aortic wall area increases due to aortic wall remodeling, a process that involves a variety of structural arterial wall alterations and is suggested mostly to occur in the early stages of atherosclerosis due to the cumulative impact of various (CV risk) factors [23, 26]. Ageing is the major determinant of structural changes arising in the walls of large arteries. With increasing age, arteries dilate and stiffen and show intimal thickening [3]. Because of the influence of sex hormones, sex is also a major contributor to differences in arterial wall dimensions between men and women [27]. Men have larger arterial wall dimensions than women. Furthermore, BMI is considered an important regulator of early abnormalities in arterial wall structure due to its relation to early signs of vascular remodeling [9]. Hence, our results appear to be biologically plausible.

In addition to aortic wall area, aortic wall thickness related to BMI. This has been reported before in healthy and CV diseased individuals and seems likely given the above-described role of BMI as regulator of early arterial wall alterations [2, 9, 10]. However, aortic wall thickness did not relate to age and sex in this young population, which is interesting as age and sex are usually considered the key drivers of atherosclerosis [6, 9, 27, 28]. Moreover, as opposed to prior studies, aortic wall thickness did not relate to any of the other studied CV risk factors. For thoracic aortic PWV, we observed a relation with SBP and DBP, which is logical given that blood pressure is one of the major contributing factors to PWV, yet we did not observe a relation between PWV and any of the other studied CV risk factors [8].

The lack of detecting associations as observed in prior studies is not surprising. Whereas prior studies comprised individuals with a large variability in age range, ethnic background and atherosclerosis burden, this study involved healthy, young individuals, within a narrow age range (25–35 years of age) and mostly of Dutch ethnicity (~90%) [5–10, 24]. In addition, our participants had a large variation in BMI as compared to the variation in blood pressure and lipid levels, which may clarify the strong association of BMI with the studied aortic characteristics as compared to the other CV risk factors. Furthermore, it is known that detrimental alterations in arterial wall structure and function are a result of a gradual development with age in combination with a cumulative exposure of the arterial wall to (un)known pathologic factors (i.e. CV risk factors) over decades, which is reflected in the strong association of these aortic characteristics with age [3, 8, 9, 24, 29]. Because of the narrow age range, the window of exposure to CV risk factors in our study population may not have been large enough to exert an effect on aortic characteristics that is visible with MRI. Another clarification for the discrepancy in results between our and prior studies is the existing heterogeneity across studies due to variations in study population, sample size, used field strength and sequences, as well as in measurement location and quantification method.

Our results warrant validation in larger cohorts. Not only may that provide further insight into the pathophysiology and key determinants of atherosclerosis development and progression in young adulthood, it may also aid in identifying high-risk groups in young adults and CV risk factors of target for preventive strategies in these individuals. Pre-clinical identification of high-risk groups in young adulthood may be realized by developing a scoring algorithm composed of aggregated CV risk factors. This algorithm may improve our ability to risk-stratify young adults and might permit early initiation of tailored therapeutic strategies aimed at modifiable CV risk factors. Despite the query whether the window of exposure to CV risk factors in young adults has been long enough to enable stratification using the above mentioned method and despite that existing algorithms assessing CV risk do not completely clarify CV risk, to date, no other biomarker has demonstrated to be of added value to current CV risk scoring algorithms [28, 30].

Strengths of our study are the meticulous random selection of young, asymptomatic general population-based sample and the combined evaluation of aortic morphology and function that may be relevant for detection of early manifestations of atherosclerosis. However, it is essential to acknowledge limitations. We did not use ECG triggering or breath holding for the 3D-T1-BB-VISTA sequence, neither did we use breath holding for the VE PWV acquisition sequences. Although this is a strength since participants did not need to follow any commands, pulsation and breathing artefacts may have introduced minor measurement errors. However, we do not think this has influenced our results since our quantification methods have previously shown to be highly reproducible and appear to be in accordance with the theory of arterial ageing as shown in previously published studies [16, 17]. Furthermore, as mentioned afore, the direction of the relations observed in the present study coincide with biological evidence and with directions of similar associations reported in prior studies. However, optimization of the 3D-T1-BB-VISTA sequence by using acceleration techniques, such as compressed sensing and motion navigators or special motion compensation methods may lead to shorter acquisition times and improved accuracy of measurement. Moreover, variation in study methodology due to the lack of a clear reference standard for measurement of MRI-derived aortic parameters limits the comparison of results across studies. Hence, the development of a standardized method of measurement and quantification of aortic MRI-parameters is required. Second, our study population mainly entailed Caucasian adults. Since atherosclerosis may exert different effects in other populations, this restricts the generalizability of our results. Third, quantification of the studied characteristics was time-consuming since it was performed semi-automatically. Hence, we only analysed a subsample of all available aortic wall data. Therefore, we cannot rule out that subtle focal lesions may have remained unnoticed. Moreover, the currently used semi-automated tool requires devoted training of observers who perform the image analysis. Hence, to improve efficiency of quantification and permit arterial wall evaluation over a larger anatomical coverage, the employment of fully automated quantification tools is merited.

## Conclusion

Variations in MRI-derived aortic wall area, wall thickness and stiffness can already be detected in young adulthood and appear to be determined by age, sex, BMI, and blood pressure.
